# Macrophage Activation Marker Soluble CD163 Associated with Fatal and Severe Ebola Virus Disease in Humans^1^

**DOI:** 10.3201/eid2502.181326

**Published:** 2019-02

**Authors:** Anita K. McElroy, Punya Shrivastava-Ranjan, Jessica R. Harmon, Roosecelis B. Martines, Luciana Silva-Flannery, Timothy D. Flietstra, Colleen S. Kraft, Aneesh K. Mehta, G. Marshall Lyon, Jay B. Varkey, Bruce S. Ribner, Stuart T. Nichol, Sherif R. Zaki, Christina F. Spiropoulou

**Affiliations:** University of Pittsburgh, Pittsburgh, Pennsylvania, USA (A.K. McElroy);; Centers for Disease Control and Prevention, Atlanta (A.K. McElroy, P. Shrivastava-Ranjan, J.R. Harmon, R.B. Martines, L Silva-Flannery, T.D. Flietstra, S.T. Nichol, S.R. Zaki, C.F. Spiropoulou);; Emory University School of Medicine, Atlanta, Georgia, USA (A.K. McElroy, C.S. Kraft, A.K. Mehta, G.M. Lyon, J.B. Varkey, B.S. Ribner)

**Keywords:** Ebola, inflammation, macrophage, MAS, HLH, Ebola virus, Ebola virus disease, sCD163, activation marker, severe disease, humans, viruses, macrophage activation syndrome, hemophagocytic lymphohistiocytosis, sCD25, CD163, T cells, hyperferritinemia, hypertriglyceridemia, zoonoses

## Abstract

Ebola virus disease (EVD) is associated with elevated cytokine levels, and hypercytokinemia is more pronounced in fatal cases. This type of hyperinflammatory state is reminiscent of 2 rheumatologic disorders known as macrophage activation syndrome and hemophagocytic lymphohistiocytosis, which are characterized by macrophage and T-cell activation. An evaluation of 2 cohorts of patients with EVD revealed that a marker of macrophage activation (sCD163) but not T-cell activation (sCD25) was associated with severe and fatal EVD. Furthermore, substantial immunoreactivity of host tissues to a CD163-specific antibody, predominantly in areas of extensive immunostaining for Ebola virus antigens, was observed in fatal cases. These data suggest that host macrophage activation contributes to EVD pathogenesis and that directed antiinflammatory therapies could be beneficial in the treatment of EVD.

Ebola virus (EBOV) disease (EVD) in humans is frequently severe and accompanied by fever, signs of endothelial dysfunction, coagulopathy, shock, and multisystem organ dysfunction. Data from nonhuman primate models and human autopsy cases suggest that EVD severity is not a direct effect of tissue damage resulting from destruction of infected cells because only foci of necrosis are observed (*1*,*2*). Therefore, a dysregulated immune response has been hypothesized to contribute to disease severity. The elevated levels of inflammatory cytokines and chemokines (e.g., interleukin [IL] 6, IL-8, macrophage inflammatory protein 1α and 1β, monocyte chemoattractant protein 1, and macrophage colony-stimulating factor) and immunomodulatory cytokines (e.g., IL-10 and IL-1 receptor antagonist) in fatal EVD cases certainly support this hypothesis (*3*–*7*). Hypercytokinemia accompanied by severe clinical disease seen in EVD is reminiscent of what has been described for macrophage activation syndrome (MAS) and hemophagocytic lymphohistiocytosis (HLH). The similarities between EVD and HLH have not gone unnoticed by other clinicians; a group in the Netherlands has published an opinion piece suggesting a connection between the 2 diseases (*8*).

HLH can occur as a primary genetic disorder or a secondary consequence of another medical condition, including infection (*9*). Secondary, virus-associated HLH is most commonly reported after Epstein-Barr virus (EBV) infection, and even though EBV infection is exceedingly common (seroprevalence in adults 80%–90%) (*10*), development of EBV-associated HLH is still a rare event, estimated at 0.4 cases/1 million population (*9*). Other hemorrhagic fever viruses, such as Crimean-Congo hemorrhagic fever virus (*11*) and dengue virus (*12*), have also been reported to trigger HLH.

MAS is considered a subgroup of HLH that is more commonly seen in patients with underlying systemic juvenile idiopathic arthritis. However, laboratory findings in both disorders are similar and include cytopenias of several cell types; elevated transaminases, soluble IL-2 receptor (sIL-2R), triglycerides, ferritin, soluble CD163 (sCD163), prothrombin time, partial thromboplastin time, and D-dimer; and low fibrinogen (*13*).

These MAS markers have been examined in patients with dengue. Elevated sCD163 and ferritin were associated with severe dengue, and sIL-2R was elevated in patients with dengue but did not distinguish between patients with severe dengue and dengue fever (*14*). Dengue-infected patients also had decreased monocyte-associated CD163 compared with healthy controls, consistent with the increase in sCD163 observed in their serum.

The proliferation and activation of macrophages and T cells and their secretion of proinflammatory cytokines has been proposed to contribute to the pathogenesis of both HLH and MAS. In addition, activated macrophages are sometimes noted to phagocytose erythrocytes, hence the term hemophagocytosis (*13*).

T-cell activation during acute EVD is significantly increased (*15*). EBOV interactions with T cells in vitro through T-cell immunoglobulin and mucin domain 1 (TIM-1) can promote a cytokine storm (*16*), and EBOV can activate macrophages in vitro though the toll-like receptor (TLR) 4 pathway (*17*). In an effort to determine if macrophage or T-cell activation–mediated mechanisms of pathogenesis (similar to those reported for MAS and HLH) could be contributing to EVD pathogenesis, we evaluated for the inflammatory markers present in MAS and HLH in 2 cohorts of patients with EVD.

## Methods

We conducted all work with human samples under approved institutional review board protocols CDC IRB 1652, CDC IRB 6341, CDC IRB 6643, and Emory IRB00076700. Before analysis, we γ-irradiated all plasma samples with 5 × 10^4^ Gy. We measured triglyceride levels using the Triglycerides Enzymatic Assay (XpressBio, https://xpressbio.com) according to the manufacturer’s instructions. We measured fibrinogen, ferritin, and sIL-2R as part of a multiplex immunoassay using methods previously described (*3*,*18*) and sCD163 using the Human CD163 Quantikine ELISA Kit (R&D Systems, https://www.rndsystems.com).

We obtained formalin-fixed paraffin-embedded sections of liver, heart, spleen, and testicle specimens from humans who died of EVD, specimens that were obtained from and previously evaluated by the Centers for Disease Control and Prevention (CDC; Atlanta, Georgia, USA) (*2*). We performed immunohistochemical stains with a mouse monoclonal antibody against CD163 (clone 10D6, dilution 1:50; Leica Biosystems, https://www.leicabiosystems.com) and polyclonal rabbit antibody against EBOV antigen (dilution 1:1,000; CDC) (*19*,*20*). We deparaffinized sections in xylene and rehydrated in a graded ethanol series. For double-stained assays, we used the EnVision G|2 Doublestain System, Rabbit/Mouse (DAB+/Permanent Red) (Dako, https://www.agilent.com/en-us/dako-products) and incubated with the CD163 monoclonal antibody and then the EBOV antibody. We performed all assays according to the manufacturers’ guidelines. We used 3,3’-diaminobenzidine (DAB) as the chromogen for the monoclonal antibody against CD163 and permanent red as the chromogen for the polyclonal antibody against EBOV. Negative control samples comprised sequential tissue sections obtained from EVD patients that were incubated with normal mouse serum stained in parallel. Also, as another control, we double-stained heart, liver, spleen, and testicle specimens from patients who died of noninfectious etiologies.

We compared biomarker levels between fatal and nonfatal cases using a previously described statistical analysis (*3*). In brief, we conducted an analysis of variance with use of the Bonferroni inequality and Bejamini and Hochberg false-discovery rate method to correct for multiple testing. Then, we performed model selection using stepwise regression to determine if biomarkers were significantly associated with death at the various time intervals. We performed posthoc Student *t*-tests for each time interval to determine statistical significance between fatal and nonfatal groups for each analyte.

## Results

We compared the laboratory features of EVD with those common in MAS and HLH, including those apart from the MAS and HLH formal diagnostic criteria (*21*–*23*). Cytopenia of erythrocytes, platelets, or neutrophils are common in both MAS and HLH (Table) (*13*). Complete blood counts (with or without differentials) have not been performed with substantial numbers of patients with EVD, with the exception of 2 reports: 1 report on patients cared for in tertiary care settings during the West Africa outbreak (*24*) and 1 report including >100 patients treated during the West Africa outbreak (*25*). Anemia to the degree seen in HLH does not appear to be common in patients with EVD. In fact, in the large cohort from West Africa, hemoconcentration, rather than anemia, was associated with fatal outcomes (*25*). Few patients with EVD have thrombocytopenia <100 × 10^3^/mL, and in the West Africa cohort, thrombocytopenia was more common in survivors. Neutropenia has been rarely seen in patients with EVD, and neutrophilia, not neutropenia, was associated with fatal outcomes in patients with EVD in West Africa. Neutrophilia could reflect the presence of a complicating secondary bacteremia that has been reported in patients with EVD (*29*). Therefore, the degrees of cytopenia seen in patients with EVD are more similar to those of MAS than HLH (Table).

**Table:** Laboratory findings of patients with MAS, HLH, or EVD and their association with fatal EVD outcomes*

Laboratory finding	MAS diagnostic value	HLH diagnostic value	EVD	Associated with fatal EVD outcome	References
Cytopenia	–	>2 cell types			
Anemia, g/dL	–	<9	Rarely	No	(*24*,*25*)
Thrombocytopenia, x 10^3^/mL	<181	<100	Sometimes	No	(*24*,*25*)
Neutropenia, cells/mL	–	<1,000	Rarely	No	(*16*,*26*)
Hyperferritinemia, ng/mL	>684	>500	Yes	Yes	(*3*,*18*)
Hypofibrinogenemia, mg/dL	<360	<150	Sometimes	Unknown	(*18*)
Hypertriglyceridemia, mg/dL	>156	>265	Sometimes	Yes	This study
sIL-2R (sCD25), U/mL	–	>2,400	Yes	No	(*18*); this study
Low or absent NK cell activity	–	–	Yes	Yes	(*27*)
Hemophagocytosis in BM, spleen, or LN	–	–	None reported		(*2*)
Soluble CD163	–	–	Yes	Yes	This study
Elevated AST/ALT, U/L	>48	–	Yes	Yes	(*28*)
Elevated D-dimer	–	–	Yes	Yes	(*3*,*18*,*28*)

*ALT, alanine aminotransferase; AST, aspartate aminotransferase; BM, bone marrow; EVD, Ebola virus disease; HLH, hemophagocytic lymphohistiocytosis; LN, lymph node; MAS, macrophage activation syndrome; NK, natural killer; sIL-2R, soluble interleukin 2 receptor; –, feature not included in disease diagnostic criteria.

**Figure 1:**
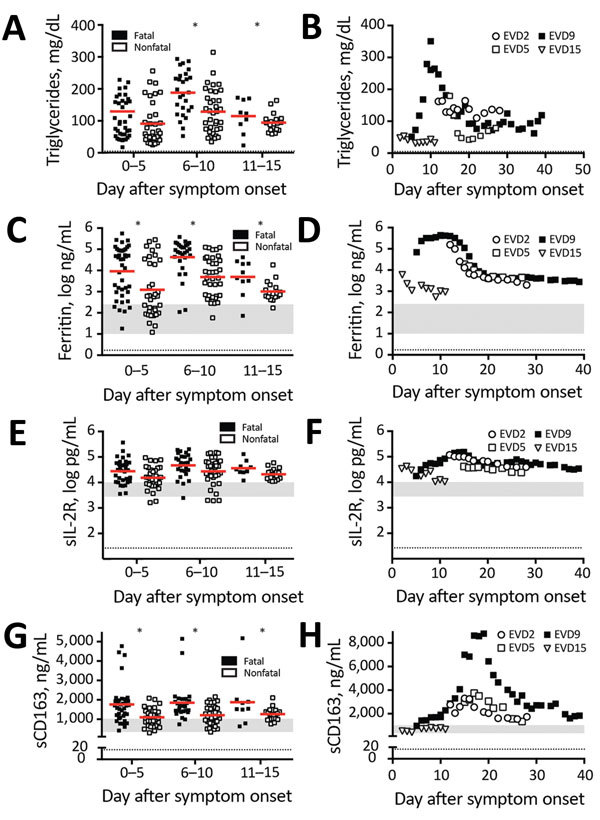
Laboratory findings of patients with EVD that are consistent with laboratory findings in patients with macrophage activation syndrome or hemophagocytic lymphohistiocytosis. A, B) Triglycerides; C, D) ferritin; E, F) sIL-2R; and G, H) sCD163. Levels were measured in the plasma of a series of 86 Sudan virus–infected patients (left column) or 4 Ebola virus–infected patients (right column). Solid horizontal lines indicate means. Gray shaded areas represent the level of the analyte detected in 10 healthy donors. Dotted lines indicate limit of detection. C) From McElroy AK, Erickson BR, Flietstra TD, Rollin PE, Nichol ST, Towner JS, et al. Ebola hemorrhagic fever: novel biomarker correlates of clinical outcome. J Infect Dis. 2014;210:558–66 (*3*); reproduced with permission. D, F) From McElroy AK, Harmon JR, Flietstra TD, Campbell S, Mehta AK, Kraft CS, et al. Kinetic analysis of biomarkers in a cohort of US patients with Ebola virus disease. Clin Infect Dis. 2016;63:460–7 (*18*); reproduced with permission. *Statistically significant difference between fatal and nonfatal cases (p<0.05). EVD, Ebola virus disease; sCD163, soluble CD163; sIL-2R, soluble interleukin 2 receptor.

A fasting hypertriglyceridemia and hypofibrinogenemia are often seen in HLH and MAS (Table) (*23*). Triglyceride levels had not been previously reported for EVD, so we measured them in plasma samples from EVD patients in 2 previously reported groups: 86 persons infected with Sudan virus (SUDV) during an outbreak in Gulu, Uganda, during 2000–2001 (*3*) (Figure 1, panel A) and 4 persons infected with EBOV treated at Emory University Hospital (Atlanta, Georgia, USA) in 2014 who survived (Figure 1, panel B) (*18*). Triglyceride levels were >250 mg/dL in severely ill patient EVD9, and in the SUDV-infected cohort, patients with fatal outcomes had significantly higher triglyceride levels, the caveat being that fasting triglyceride levels could not be determined. However, triglyceride levels in these patients do correlate with both severe and fatal disease. In a previous study, fibrinogen levels were measured in an EBOV cohort (*18*) and reported to be low in all patients, but an association with severity was not demonstrated.

Patients with MAS or HLH often have elevated ferritin (Table). As has been previously reported (*3*,*18*), ferritin levels were well above the MAS and HLH criterion in most patients with EVD (Table; Figure 1, panels C, D). Furthermore, high ferritin levels were associated with fatal outcomes in SUDV-infected patients. In fact, exceptionally high ferritin levels (>10,000 ng/mL), which were seen in many patients with EVD, have been suggested to be an independent predictor of HLH with high sensitivity and specificity (*21*).

sIL-2R, also known as soluble CD25, a marker of T-cell activation, has been reported to be elevated in HLH and MAS (Table). We also assessed this marker in the same 2 patient cohorts that we previously described. Many SUDV-infected patients had sIL-2R levels well above the upper limit of normal, but the level did not correlate with fatal outcome (Figure 1, panel E); all EBOV-infected patients had elevated levels of sIL-2R, regardless of disease severity, as previously reported (Figure 1, panel F) (*18*).

Natural killer [NK] cell activity and hemophagocytosis are often altered in HLH and MAS. Flow cytometric evaluation of NK cell populations typically reveals diminished cell numbers and function, and pathologic analysis of bone marrow biopsies or autopsy specimens might reveal evidence of hemophagocytosis. Advanced flow cytometric evaluations of peripheral blood from EBOV-infected patients were published after the West Africa outbreak (*30*,*31*), and a study reported lower numbers of total NK cells in patients with fatal cases of EVD (*27*). Although the activation status of the NK cells detected did not differ between those who survived and those who died, lower numbers of cells would be consistent with overall decreased NK cell activity in patients with fatal EVD. The lack of human bone marrow biopsy and autopsy specimens limited our ability to look for hemophagocytosis in EVD, but a detailed pathology study did not indicate any signs of hemophagocytosis in spleen, lymph node, or bone marrow samples from patients with EVD, although viral inclusions were noted in these tissues, specifically in cells of the mononuclear phagocytic system (*2*).

Measurement of sCD163, the haptoglobin-hemoglobin scavenger receptor and a marker of macrophage activation, has been examined in several studies of MAS and HLH (*32*–*34*). CD163 expression was reported on hemophagocytic macrophages in the skin of patients with HLH, and high serum levels have been noted in patients with HLH and MAS. sCD163 has also been associated with disease severity in hemorrhagic fever with renal syndrome (which occurs after hantavirus infection) and dengue hemorrhagic fever (*14*,*35*). We assessed the levels of sCD163 in the blood of patients of the SUDV and EBOV cohorts. In SUDV-infected patients, the sCD163 level was elevated (Figure 1, panel G), and high sCD163 concentration was associated with fatal outcome. The sCD163 level was high in a severely affected EBOV-infected patient (EVD9) who would not have survived without extracorporeal supportive care (Figure 1, panel H). In this patient, the peak in sCD163 occurred 19 days after symptom onset, at a point when the viremia was well controlled, having peaked at day 10 and declined thereafter (*18*). In fact, a strong negative correlation between viral load and sCD163 level was evident in EVD9 (Spearman correlation coefficient −0.9059). This patient remained critically ill after viral load control, suggesting that the inflammatory response was a substantial contributor to disease manifestation.

Two other laboratory findings often reported in HLH and MAS are elevated transaminases and D-dimers (Table) (*13*). Aspartate aminotransferase and D-dimers are elevated in patients with EVD, and elevated levels of these analytes are associated with fatal outcome (*3*,*28*).

**Figure 2:**
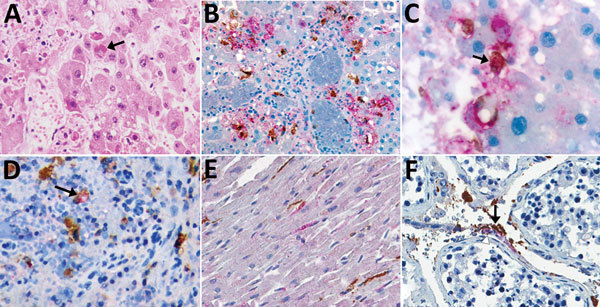
Immunohistochemical stains of tissue from patients with fatal cases of Ebola virus (EBOV) disease showing EBOV (red) and CD163 (brown) antigens. A) Hematoxylin and eosin stain of liver showing hepatocellular necrosis with intracytoplasmic eosinophilic inclusions (arrow). B) EBOV antigens in hepatocytes and CD163 antigens in macrophages. C) High magnification image of double immunohistochemical staining of liver tissue showing colocalization of EBOV and CD163 antigens in macrophage (arrow). D) Colocalization of EBOV and CD163 antigen in macrophage of spleen (arrow). E) Staining of EBOV and interstitial macrophages (CD163) in heart. EBOV found in some cardiomyocytes. F) EBOV and CD163 antigen in endothelial cells (arrowhead) and macrophages of testis (arrow). Original magnification ×20 (A, B, D, E, F); ×63 (C).

Given these laboratory findings suggesting an association between macrophage activation and EVD pathogenesis, we performed an analysis for CD163 protein expression with tissues from patients with fatal EVD. Liver sections from patients with fatal EVD showed hepatocyte necrosis, often with minimal inflammation (Figure 2, panel A). Mild-to-moderate small-droplet steatosis and Kupffer cell hyperplasia were also seen. Hepatocytes had characteristic intracytoplasmic eosinophilic inclusions, which were predominantly found in periportal zones and surrounding areas of necrosis.

**Figure 3:**
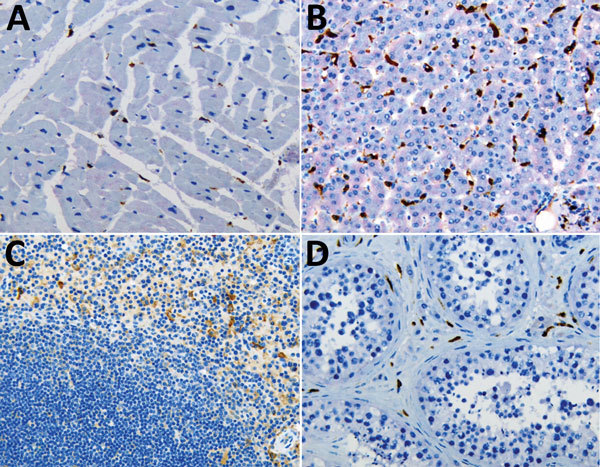
Double immunohistochemical staining of Ebola virus (red) and CD163 antigen (brown) in tissues of patients who died of noninfectious causes. CD163 antigens in macrophages of heart (A), liver (Kupffer cells) (B), spleen (C), and testicle (D). Original magnification ×20.

We performed double-stained immunoassays to assess for colocalization of EBOV and CD163. Double staining confirmed the presence of viral antigens predominantly within hepatocytes and macrophages (Figure 2, panels B–D), as well as increased levels of CD163 in association with viral antigens. The CD163 immunostaining of the myocardium from a fatal case of EVD did not differ substantially from that of a patient who died from a noninfectious cause (Figure 2, panel E; Figure 3, panel A). Of note, CD163-positive immunostaining in the interstitial space of the testes colocalized with viral antigen (Figure 2, panel F). Tissue-resident macrophages are known to exhibit CD163 staining, so several tissue samples from patients who died of a noninfectious cause were examined to have a baseline for comparison. CD163 staining identified Kupffer cells of the liver (Figure 3, panel B), tissue-resident macrophages of the myocardium (Figure 3, panel A) and spleen (Figure 3, panel C), and macrophages in the interstitial space of the testes (Figure 3, panel D). In summary, tissues from patients with fatal EVD showed increased CD163-positive macrophages near the areas of extensive immunostaining for EBOV antigens.

## Discussion

Both HLH and MAS are inflammatory syndromes that are characterized by fever, hypercytokinemia, liver dysfunction, and coagulopathy. EVD shows some striking similarities to these 2 disorders, suggesting a common underlying mechanism of pathology. T-cell activation and proliferation, evidenced by elevated sIL-2R (sCD25) levels, are hypothesized to be responsible for the bulk of the hypercytokinemia in HLH and MAS. CD25 is upregulated on activated T cells, and sCD25 levels correlate with membrane-bound CD25 levels on lymphocytes (*36*). The interaction between EBOV and TIM-1 on the surface of T cells has been demonstrated to lead to nonspecific T-cell activation and elevation of proinflammatory cytokines (*16*). However, TIM proteins are expressed on other cell types, so TIM-mediated signaling with cells other than T cells could be involved in EVD. Because all of the EVD patients we studied had elevated levels of sCD25 and T-cell activation has been reported in patients with EVD (*15*), hypercytokinemia could result from T-cell activation through a nonspecific component. However, a substantial amount of data suggests that T-cell function (presumably antigen-specific T-cell function) is critical for virus control and host survival (*37*). In addition, no correlation between sCD25 level and disease severity or outcome was evident; thus, the elevation of sCD25 might simply reflect the fact that T cells are activated after infection. Therefore, any therapeutic attempt to modulate T-cell activity to improve patient outcome must take into account the fact that different populations of T cells within the host could be simultaneously deleterious and beneficial.

In healthy persons, the ferritin level is 10–250 ng/mL and used clinically as a marker of iron storage (*26*). Ferritin is also known as an acute-phase reactant that is elevated nonspecifically in the context of inflammation. In HLH and MAS, ferritin levels can be markedly high. Hyperferritinemia was observed in patients with EVD, where ferritin levels were as high as 1 × 10^5^ ng/mL, which is 3 logs of magnitude over the upper limit of normal. In HLH and MAS, the source of ferritin causing the extremely high blood levels is thought to be the activated macrophage population (*38*,*39*). In EVD, the liver could also be the source of the ferritin, as has been noted in animal models (*40*).

Elevations in serum triglycerides can occur in response to inflammation; this rise in concentration is thought to be mediated by cytokine-dependent increases in hepatic secretion (*41*). The elevated triglycerides seen in severe and fatal cases of EVD would be consistent with this finding, considering these same patients have marked increases in proinflammatory cytokines. Therefore, the finding of elevated triglycerides probably represents a response to rather than an initiator of pathology.

In contrast with the conflicting data on T-cell activation in EVD, the data for a pathogenic role of macrophages is more convincing. Elevated levels of macrophage activation marker sCD163 were seen in all patients with EVD, and this marker was associated with both disease severity and fatal outcome. Elevated levels of sCD163 in disease pathogenesis have also been reported for dengue virus and hantavirus infections (*14*,*35*), and sCD163 was an independent predictor of all-cause mortality in HIV-infected patients (*42*).

CD163 is shed from the surface of activated macrophages or monocytes after stimulation of surface but not intracellular TLRs (*43*). This shed sCD163 can then bind hemoglobin, an activity theorized to be an innate immune signaling mechanism to help combat microbial pathogens by scavenging available iron. In vivo endotoxin studies in humans have shown that surface CD163 is rapidly replaced on monocytes within 24 hours after lipopolysaccharide-mediated TLR-4 stimulation and CD163 shedding (*44*). Therefore, that we found elevated levels of sCD163 and abundant CD163-positive macrophages in the liver surrounding areas with extensive viral antigen is not surprising. Liver macrophages include mostly Kupffer cells, which are usual constituents of the liver, but during inflammation, monocytes are recruited from the periphery that can differentiate into macrophages in this tissue (*45*). Kupffer cells express various markers and are thought to have various functions. CD163 is classically considered a reparative marker of an M2 type of macrophage, but this marker is not definitive because inflammatory macrophages can also express CD163 (*46*). The finding of high levels of sCD163 in fatal cases of EVD and of CD163 immunostaining in association with viral antigen in the tissues of fatal cases suggests that virally mediated activation of macrophages contributes to EVD pathogenesis in vivo.

Activation of macrophages or monocytes after in vitro EBOV infection leads to massive cytokine secretion (*47*,*48*), which could also explain the hypercytokinemia that has been observed in patients with EVD. Pathogenic EBOVs and not nonpathogenic EBOV (e.g., Reston) were shown to activate macrophages by TLR-4 (*17*), and treatment of mice with a TLR-4 antagonist improved clinical scores, decreased inflammatory responses, and increased the survival of EBOV-infected mice (*49*). These in vitro and animal model data combined with the primary human data we present together provide further evidence that not only does EBOV infection activate macrophages in vivo, but this activation also plays a direct role in the pathogenesis of the virus.

A limitation of our study is that the EBOV cohort of patients treated at Emory University Hospital received several different therapeutic interventions (*18*). Whether these interventions affected the measured parameters is unknown; however, we noted concordance of data between patients with fatal cases of SUDV not treated with therapeutics and EVD9, the patient with severe EBV given therapeutic treatments.

Although EVD does not appear to trigger the development of MAS or HLH, the similarities of their inflammatory profile suggest that some of the therapeutic interventions that have shown success in treating HLH or MAS could be beneficial in treating EVD as well. Corticosteroids, etoposide, and cyclosporine A are mainstays for treatment of HLH, and directed biologic therapies are being assessed. A search of ClinicalTrials.gov reveals ongoing and completed studies of many novel inflammation-targeting biologics for use in either MAS or HLH: blockers of interferon-γ signaling (trial nos. NCT03311854, NCT03312751, NCT01818492, and NCT03311854), blockers of IL-6 (NCT02007239), blockers of IL-1 (NCT02780583), and antithymocyte globulin (NCT01104025). All of these therapies target inflammatory mediators or, in the case of antithymocyte globulin, T cells because T cells are thought to play a role in the pathogenesis of these inflammatory disorders. Given the acute nature of EVD, we hypothesize that targeting the specific types of inflammation seen in EVD would improve patient outcomes. The TLR-4 signaling that leads to macrophage activation is another potential target for host-directed immunotherapeutics. Clinical trials in humans are underway for biologics that block TLR-4, IL-6, IL-8, IL-1, and tumor necrosis factor α for several different disease conditions. Because all of these cytokines are elevated in EVD, we advise evaluation of these therapies in EVD. In addition, the many immunotherapeutics that modulate T-cell function, both inhibitors (e.g., IL-2 blockers) and activators (e.g., programmed cell death 1 and cytotoxic T-lymphocyte–associated protein 4 antibodies), could also be evaluated as therapeutics. Regardless of which therapeutic modality is chosen, a plethora of host immunomodulatory therapies are available and should be evaluated in nonhuman primate animal models of EVD. In fact, as the marker sCD163 indicates, inflammation can persist, even after viral load control, so improving patient outcomes will probably require both directed antiviral therapies early in the disease course and carefully timed host-directed antiinflammatory therapies.

## References

[1] Geisbert TW, Young HA, Jahrling PB, Davis KJ, Larsen T, Kagan E, et al. Pathogenesis of Ebola hemorrhagic fever in primate models: evidence that hemorrhage is not a direct effect of virus-induced cytolysis of endothelial cells. Am J Pathol. 2003;163:2371–82. 10.1016/S0002-9440(10)63592-4 14633609 PMC1892396

[2] Martines RB, Ng DL, Greer PW, Rollin PE, Zaki SR. Tissue and cellular tropism, pathology and pathogenesis of Ebola and Marburg viruses. J Pathol. 2015;235:153–74. 10.1002/path.4456 25297522

[3] McElroy AK, Erickson BR, Flietstra TD, Rollin PE, Nichol ST, Towner JS, et al. Ebola hemorrhagic Fever: novel biomarker correlates of clinical outcome. J Infect Dis. 2014;210:558–66. 10.1093/infdis/jiu088 24526742 PMC4172044

[4] Wauquier N, Becquart P, Padilla C, Baize S, Leroy EM. Human fatal zaire ebola virus infection is associated with an aberrant innate immunity and with massive lymphocyte apoptosis. PLoS Negl Trop Dis. 2010;4:e837. 10.1371/journal.pntd.0000837 20957152 PMC2950153

[5] Hutchinson KL, Rollin PE. Cytokine and chemokine expression in humans infected with Sudan Ebola virus. J Infect Dis. 2007;196(Suppl 2):S357–63. 10.1086/520611 17940971

[6] Baize S, Leroy EM, Georges AJ, Georges-Courbot MC, Capron M, Bedjabaga I, et al. Inflammatory responses in Ebola virus-infected patients. Clin Exp Immunol. 2002;128:163–8. 10.1046/j.1365-2249.2002.01800.x 11982604 PMC1906357

[7] Villinger F, Rollin PE, Brar SS, Chikkala NF, Winter J, Sundstrom JB, et al. Markedly elevated levels of interferon (IFN)-gamma, IFN-alpha, interleukin (IL)-2, IL-10, and tumor necrosis factor-alpha associated with fatal Ebola virus infection. J Infect Dis. 1999;179(Suppl 1):S188–91. 10.1086/514283 9988183

[8] van der Ven AJ, Netea MG, van der Meer JW, de Mast Q. Ebola virus disease has features of hemophagocytic lymphohistiocytosis syndrome. Front Med (Lausanne). 2015;2:4. 10.3389/fmed.2015.00004 25699258 PMC4316785

[9] George MR. Hemophagocytic lymphohistiocytosis: review of etiologies and management. J Blood Med. 2014;5:69–86. 10.2147/JBM.S46255 24966707 PMC4062561

[10] Dowd JB, Palermo T, Brite J, McDade TW, Aiello A. Seroprevalence of Epstein-Barr virus infection in U.S. children ages 6-19, 2003-2010. PLoS One. 2013;8:e64921. 10.1371/journal.pone.0064921 23717674 PMC3661547

[11] Tasdelen Fisgin N, Fisgin T, Tanyel E, Doganci L, Tulek N, Guler N, et al. Crimean-Congo hemorrhagic fever: five patients with hemophagocytic syndrome. Am J Hematol. 2008;83:73–6. 10.1002/ajh.20969 17597475

[12] Wan Jamaludin WF, Periyasamy P, Wan Mat WR, Abdul Wahid SF. Dengue infection associated hemophagocytic syndrome: Therapeutic interventions and outcome. J Clin Virol. 2015;69:91–5. 10.1016/j.jcv.2015.06.004 26209387

[13] Grom AA, Horne A, De Benedetti F. Macrophage activation syndrome in the era of biologic therapy. Nat Rev Rheumatol. 2016;12:259–68. 10.1038/nrrheum.2015.179 27009539 PMC5851441

[14] Ab-Rahman HA, Rahim H, AbuBakar S, Wong PF. Macrophage activation syndrome-associated markers in severe dengue. Int J Med Sci. 2016;13:179–86. 10.7150/ijms.13680 26941578 PMC4773282

[15] McElroy AK, Akondy RS, Davis CW, Ellebedy AH, Mehta AK, Kraft CS, et al. Human Ebola virus infection results in substantial immune activation. Proc Natl Acad Sci U S A. 2015;112:4719–24. 10.1073/pnas.1502619112 25775592 PMC4403189

[16] Younan P, Iampietro M, Nishida A, Ramanathan P, Santos RI, Dutta M, et al. Ebola virus binding to TIM-1 on T lymphocytes induces a cytokine storm. MBio. 2017;8:e00845-17. 10.1128/mBio.00845-17 28951472 PMC5615193

[17] Olejnik J, Forero A, Deflubé LR, Hume AJ, Manhart WA, Nishida A, et al. Ebolaviruses associated with differential pathogenicity induce distinct host responses in human macrophages. J Virol. 2017;91:e00179-17. 10.1128/JVI.00179-17 28331091 PMC5432886

[18] McElroy AK, Harmon JR, Flietstra TD, Campbell S, Mehta AK, Kraft CS, et al. Kinetic analysis of biomarkers in a cohort of US patients with Ebola virus disease. Clin Infect Dis. 2016;63:460–7. 10.1093/cid/ciw334 27353663 PMC4967605

[19] Ksiazek TG, Rollin PE, Jahrling PB, Johnson E, Dalgard DW, Peters CJ. Enzyme immunosorbent assay for Ebola virus antigens in tissues of infected primates. J Clin Microbiol. 1992;30:947–50. 1572982 10.1128/jcm.30.4.947-950.1992 PMC265191

[20] Zaki SR, Shieh WJ, Greer PW, Goldsmith CS, Ferebee T, Katshitshi J, et al. A novel immunohistochemical assay for the detection of Ebola virus in skin: implications for diagnosis, spread, and surveillance of Ebola hemorrhagic fever. Commission de Lutte contre les Epidémies à Kikwit. J Infect Dis. 1999;179(Suppl 1):S36–47. 10.1086/514319 9988163

[21] Lehmberg K, Ehl S. Diagnostic evaluation of patients with suspected haemophagocytic lymphohistiocytosis. Br J Haematol. 2013;160:275–87. 10.1111/bjh.12138 23206255

[22] Henter JI, Horne A, Aricó M, Egeler RM, Filipovich AH, Imashuku S, et al. HLH-2004: Diagnostic and therapeutic guidelines for hemophagocytic lymphohistiocytosis. Pediatr Blood Cancer. 2007;48:124–31. 10.1002/pbc.21039 16937360

[23] Ravelli A, Minoia F, Davì S, Horne A, Bovis F, Pistorio A, et al.; Paediatric Rheumatology International Trials Organisation; Childhood Arthritis and Rheumatology Research Alliance; Pediatric Rheumatology Collaborative Study Group; Histiocyte Society. 2016 classification criteria for macrophage activation syndrome complicating systemic juvenile idiopathic arthritis: a European League Against Rheumatism/American College of Rheumatology/Paediatric Rheumatology International Trials Organisation collaborative initiative. Arthritis Rheumatol. 2016;68:566–76. 10.1002/art.39332 26314788

[24] Uyeki TM, Mehta AK, Davey RT Jr, Liddell AM, Wolf T, Vetter P, et al.; Working Group of the U.S.–European Clinical Network on Clinical Management of Ebola Virus Disease Patients in the U.S. and Europe. Clinical management of Ebola virus disease in the United States and Europe. N Engl J Med. 2016;374:636–46. 10.1056/NEJMoa1504874 26886522 PMC4972324

[25] Hunt L, Gupta-Wright A, Simms V, Tamba F, Knott V, Tamba K, et al. Clinical presentation, biochemical, and haematological parameters and their association with outcome in patients with Ebola virus disease: an observational cohort study. Lancet Infect Dis. 2015;15:1292–9. 10.1016/S1473-3099(15)00144-9 26271406

[26] Finch CA, Bellotti V, Stray S, Lipschitz DA, Cook JD, Pippard MJ, et al. Plasma ferritin determination as a diagnostic tool. West J Med. 1986;145:657–63. 3541387 PMC1307110

[27] Cimini E, Viola D, Cabeza-Cabrerizo M, Romanelli A, Tumino N, Sacchi A, et al. Different features of Vδ2 T and NK cells in fatal and non-fatal human Ebola infections. PLoS Negl Trop Dis. 2017;11:e0005645. 10.1371/journal.pntd.0005645 28558022 PMC5472323

[28] Rollin PE, Bausch DG, Sanchez A. Blood chemistry measurements and D-Dimer levels associated with fatal and nonfatal outcomes in humans infected with Sudan Ebola virus. J Infect Dis. 2007;196(Suppl 2):S364–71. 10.1086/520613 17940972

[29] Kreuels B, Wichmann D, Emmerich P, Schmidt-Chanasit J, de Heer G, Kluge S, et al. A case of severe Ebola virus infection complicated by gram-negative septicemia. N Engl J Med. 2014;371:2394–401. 10.1056/NEJMoa1411677 25337633

[30] Lüdtke A, Ruibal P, Becker-Ziaja B, Rottstegge M, Wozniak DM, Cabeza-Cabrerizo M, et al. Ebola virus disease is characterized by poor activation and reduced levels of circulating CD16^+^ monocytes. J Infect Dis. 2016;214(suppl 3):S275–80. 10.1093/infdis/jiw260 27521367

[31] Ruibal P, Oestereich L, Lüdtke A, Becker-Ziaja B, Wozniak DM, Kerber R, et al. Unique human immune signature of Ebola virus disease in Guinea. Nature. 2016;533:100–4. 10.1038/nature17949 27147028 PMC4876960

[32] Bleesing J, Prada A, Siegel DM, Villanueva J, Olson J, Ilowite NT, et al. The diagnostic significance of soluble CD163 and soluble interleukin-2 receptor α-chain in macrophage activation syndrome and untreated new-onset systemic juvenile idiopathic arthritis. Arthritis Rheum. 2007;56:965–71. 10.1002/art.22416 17328073

[33] Schaer DJ, Schleiffenbaum B, Kurrer M, Imhof A, Bächli E, Fehr J, et al. Soluble hemoglobin-haptoglobin scavenger receptor CD163 as a lineage-specific marker in the reactive hemophagocytic syndrome. Eur J Haematol. 2005;74:6–10. 10.1111/j.1600-0609.2004.00318.x 15613100

[34] Santos-Arroyo A, Barrera-Llaurador J, Sánchez JE, Martín-García R, Sánchez JL. Role of skin biopsies in the diagnosis of hemophagocytic lymphohistiocytosis. Am J Dermatopathol. 2017;39:e86–9. 10.1097/DAD.0000000000000825 28178007

[35] Wang J, Guo W, Du H, Yu H, Jiang W, Zhu T, et al. Elevated soluble CD163 plasma levels are associated with disease severity in patients with hemorrhagic fever with renal syndrome. PLoS One. 2014;9:e112127. 10.1371/journal.pone.0112127 25392926 PMC4230986

[36] Bogner MP, Voss SD, Bechhofer R, Hank JA, Roper M, Poplack D, et al. Serum CD25 levels during interleukin-2 therapy: dose dependence and correlations with clinical toxicity and lymphocyte surface sCD25 expression. J Immunother (1991). 1992;11:111–8. 10.1097/00002371-199202000-00005 1571333

[37] McElroy AK, Mühlberger E, Muñoz-Fontela C. Immune barriers of Ebola virus infection. Curr Opin Virol. 2018;28:152–60. 10.1016/j.coviro.2018.01.010 29452995 PMC5886007

[38] Wormsbecker AJ, Sweet DD, Mann SL, Wang SY, Pudek MR, Chen LY. Conditions associated with extreme hyperferritinaemia (>3000 μg/L) in adults. Intern Med J. 2015;45:828–33. 10.1111/imj.12768 25851400

[39] Cohen LA, Gutierrez L, Weiss A, Leichtmann-Bardoogo Y, Zhang DL, Crooks DR, et al. Serum ferritin is derived primarily from macrophages through a nonclassical secretory pathway. Blood. 2010;116:1574–84. 10.1182/blood-2009-11-253815 20472835

[40] Naz N, Moriconi F, Ahmad S, Amanzada A, Khan S, Mihm S, et al. Ferritin L is the sole serum ferritin constituent and a positive hepatic acute-phase protein. Shock. 2013;39:520–6. 10.1097/SHK.0b013e31829266b9 23524846

[41] Feingold KR, Hardardóttir I, Grunfeld C. Beneficial effects of cytokine induced hyperlipidemia. Z Ernahrungswiss. 1998;37(Suppl 1):66–74. 9558731

[42] Knudsen TB, Ertner G, Petersen J, Møller HJ, Moestrup SK, Eugen-Olsen J, et al. Plasma soluble CD163 level independently predicts all-cause mortality in HIV-1–infected individuals. J Infect Dis. 2016;214:1198–204. 10.1093/infdis/jiw263 27354366

[43] Weaver LK, Hintz-Goldstein KA, Pioli PA, Wardwell K, Qureshi N, Vogel SN, et al. Pivotal advance: activation of cell surface Toll-like receptors causes shedding of the hemoglobin scavenger receptor CD163. J Leukoc Biol. 2006;80:26–35. 10.1189/jlb.1205756 16799153

[44] Hintz KA, Rassias AJ, Wardwell K, Moss ML, Morganelli PM, Pioli PA, et al. Endotoxin induces rapid metalloproteinase-mediated shedding followed by up-regulation of the monocyte hemoglobin scavenger receptor CD163. J Leukoc Biol. 2002;72:711–7. 12377940

[45] Sun YY, Li XF, Meng XM, Huang C, Zhang L, Li J. Macrophage phenotype in liver injury and repair. Scand J Immunol. 2017;85:166–74. 10.1111/sji.12468 27491503

[46] Barros MH, Hauck F, Dreyer JH, Kempkes B, Niedobitek G. Macrophage polarisation: an immunohistochemical approach for identifying M1 and M2 macrophages. PLoS One. 2013;8:e80908. 10.1371/journal.pone.0080908 24260507 PMC3829941

[47] Gupta M, Mahanty S, Ahmed R, Rollin PE. Monocyte-derived human macrophages and peripheral blood mononuclear cells infected with ebola virus secrete MIP-1α and TNF-α and inhibit poly-IC-induced IFN-α in vitro. Virology. 2001;284:20–5. 10.1006/viro.2001.0836 11352664

[48] Ströher U, West E, Bugany H, Klenk HD, Schnittler HJ, Feldmann H. Infection and activation of monocytes by Marburg and Ebola viruses. J Virol. 2001;75:11025–33. 10.1128/JVI.75.22.11025-11033.2001 11602743 PMC114683

[49] Younan P, Ramanathan P, Graber J, Gusovsky F, Bukreyev A. The toll-like receptor receptor 4 antagonist eritoran protects mice from lethal filovirus challenge. MBio. 2017;8:e00226-17. 10.1128/mBio.00226-17 28442605 PMC5405229

